# Integrating molecular diagnostics for early prostate cancer detection

**DOI:** 10.18632/oncoscience.620

**Published:** 2025-05-26

**Authors:** Pankaja B. Umarane, R.B. Nerli, Saniya Chaman Malik

**Affiliations:** ^1^Department of Urology, KLES Dr. Prabhakar Kore Hospital and MRC, Belgaum, Karnataka, India; ^2^Department of Urology, JN Medical College, KLE Academy of Higher Education and Research (Deemed-to-be-University), JNMC Campus, Belagavi, Karnataka 590010, India

**Keywords:** prostate cancer, PCR-RFLP, genetic biomarkers, molecular diagnostics, genes

## Abstract

Introduction: Prostate cancer (PCa) is one of the most common malignancies in men and accurate diagnostic tools are needed for early detection and risk stratification. Standard diagnostic modalities have limitations including low specificity, overdiagnosis, and procedural invasiveness. We investigate the utility of molecular diagnostics, restriction fragment length polymorphism (RFLP) for identifying mutations in genes that predispose to PCa.

Methods: The present prospective case-control study included 136 participants (66 cases and 70 controls). DNA was extracted for the evaluation of specific BRCA1, BRCA2, HOXB13, RNASEL, and ELAC2 single nucleotide polymorphisms (SNPs) using PCR-RFLP.

Result: The association of BRCA2 (rs80359550) and HOXB13 (rs9900627) mutations with the risk of developing PCa was statistically significant (*p* < 0.0001 and *p* = 0.0139, respectively) and the odds ratios confirmed a strong genetic susceptibility.

Discussion: Our findings further underscore the relevance of RFLP-based genotyping as an affordable substitute for NGS, in light of limited accessibility in many resource-limited settings.

Conclusions: Integrating genetic, molecular, or imaging readouts with additional imaging modalities, such as mpMRI offers opportunities for improved diagnostic accuracy and conceivable tailored treatment approaches. Larger multiethnic studies are needed to confirm these findings and define a genetic screening protocol for PCa.

## INTRODUCTION

Prostate cancer is among the most common cancer types in men, and the incidence continues to rise globally. Prompt and accurate identification is critical for optimal patient management, directing treatment decisions, and minimizing unnecessary testing [1]. Clinically, prostate cancer is diagnosed through non-invasive screening techniques such as prostate-specific antigen (PSA) testing and digital rectal examination (DRE), with confirmation through transrectal ultrasound (TRUS) guided biopsy and histological evaluation [2]. Although these methods represent the current standard of care, they are not without limitations, including low specificity, the potential for overdiagnosis, and the associated complications of invasive biopsy [3].

Contributions of molecular diagnostics and next-generation sequencing being a new era of prostate cancer testing for Novel genetic biomarkers, like BRCA1/2, HOXB13, ATM, CHEK2, and DNA mismatch repair (MMR) genes, provide insight into inherited risk, disease progression, and targeted therapies [4]. Moreover, RNA-based markers such as PCA3 and TMPRSS2-ERG fusion, and epigenetic changes including DNA methylation of GSTP1 and APC genes offer increased diagnostic accuracy and risk stratification [5]. Recent advances using liquid biopsy approaches, such as profiling circulating tumor DNA (ctDNA), exosomal RNA, and other non-invasive biomarkers, have emerged as appealing alternatives to traditional tissue biopsies, enabling real-time disease monitoring with minimal inconvenience to the patient [6].

While Next Generation Sequencing (NGS) has been touted as the gold standard of comprehensive genomic profiling due to its ability to detect a multitude of different genetic mutations, its high costs along with the sophisticated technology needed for infrastructure restrict its availability, especially in lower resource settings [7, 8]. In this regard, cheaper methods like Restriction Fragment Length Polymorphism (RFLP) analysis have surfaced as effective techniques for detecting genetic mutations associated with prostate cancer. RFLP is a simple and inexpensive molecular technique that can detect single nucleotide polymorphisms (SNPs) as well as other cancer-associated genetic variants linked to prostate cancer. Even though RFLP lacks the comprehensive detail provided by NGS, it is still quite practical and useful for preliminary genetic testing in resource limited environments [9]. This study examines the mutations in BRCA2 and HOXB13 which are some of the best described genes associated with hereditary prostate cancer, emphasizing the rationale of why RFLP could aid in the early diagnosis of patients in regions lacking NGS capabilities.

Each of these uses of genomic sequencing expands on the limits of traditional biopsy modalities involving invasive procedures and a significant risk of sampling error by allowing the genomic characterization of the totality of the disease at a specific moment in time and in different microenvironments, as well as providing a genetic-therapeutic predictive index of the tumor biology [10]. Despite the fact that the biopsy has long been considered the gold standard for histopathological verification of prostate cancer, the integration of NGS-driven biomarkers with imaging modalities such as multiparametric MRI (mpMRI) and positron emission tomography (PET) can greatly enhance the precision that can be obtained from any diagnostic camera [11].

In this article, we explore the changing identity of prostate cancer diagnosis beneath the conventional paradigm of biopsy-based evaluations and highlight the promise of restriction fragment length polymorphism (RFLP) in accelerating early diagnosis. Combining NGS and RFLP with imaging modalities such as multiparametric MRI (mpMRI) empowers clinicians to enhance diagnostic precision, reduce the need for invasive procedures, and personalize treatment strategies for improved patient outcomes.

## RESULTS

Comparative analysis of sociodemographic and clinical characteristics between prostate cancer cases (*n* = 66) and a control (*n* = 70) group Data are shown as n (%) (Table 1). Prostate cancer is generally diagnosed at older ages and the mean age at diagnosis was significantly higher among cases (57.47 ± 8.94 years) than among controls (51.27 ± 8.60 years) (*p* = 0.0001). Men diagnosed with prostate cancer shows a significantly elevated PSA level (111.06 ± 214.26) compared to controls (2.80 ± 1.10) with a highly significant *p*-value (< 0.0001), further exemplifying the use of PSA as a valuable diagnostic parameter. Furthermore, based on the score on the Gleason score, one of the mortality indexes, most cases fall into the moderate range (5–6) (63.64%). Family history of prostate cancer among cases (37.88%) compared to controls (5.71%) (*p* < 0.0001)) indicating a clear genetic predisposition. These findings underscore the importance of risk factors for prostate cancer, such as older age, higher PSA levels, and family history, which could improve early detection and help to inform risk stratification.

**Table 1:** Sociodemographic analysis of the patients

Total number of patients						
Parameters	Cases (*n* = 66)	%	Controls (*n* = 70)	%	*p*-value	95% CI
**Age at diagnosis**						
Mean ± SD	57.47 ± 8.94		51.27 ± 8.60		0.0001	−9.17 to −3.22
40–50	14	21.21	20	28.57		
51–60	24	36.36	20	28.57		
61–70	20	30.31	13	18.57		
>70	8	12.12	17	24.29		
**PSA**						
Mean ± SD	111.06 ± 214.26		2.80 ± 1.10		< 0.0001	−158.89 to −57.6
<10	0	0	70	100		
10–50	36	54.55	0	0		
51–100	20	30.3	0	0		
>100	10	15.15	0	0		
**Gleasons score**						
2–4	24	36.36	0	0		
5–6	42	63.64	0	0		
Family history	25	37.88	4	5.71	< 0.0001	18.74 to 44.72

**Figure 1:**
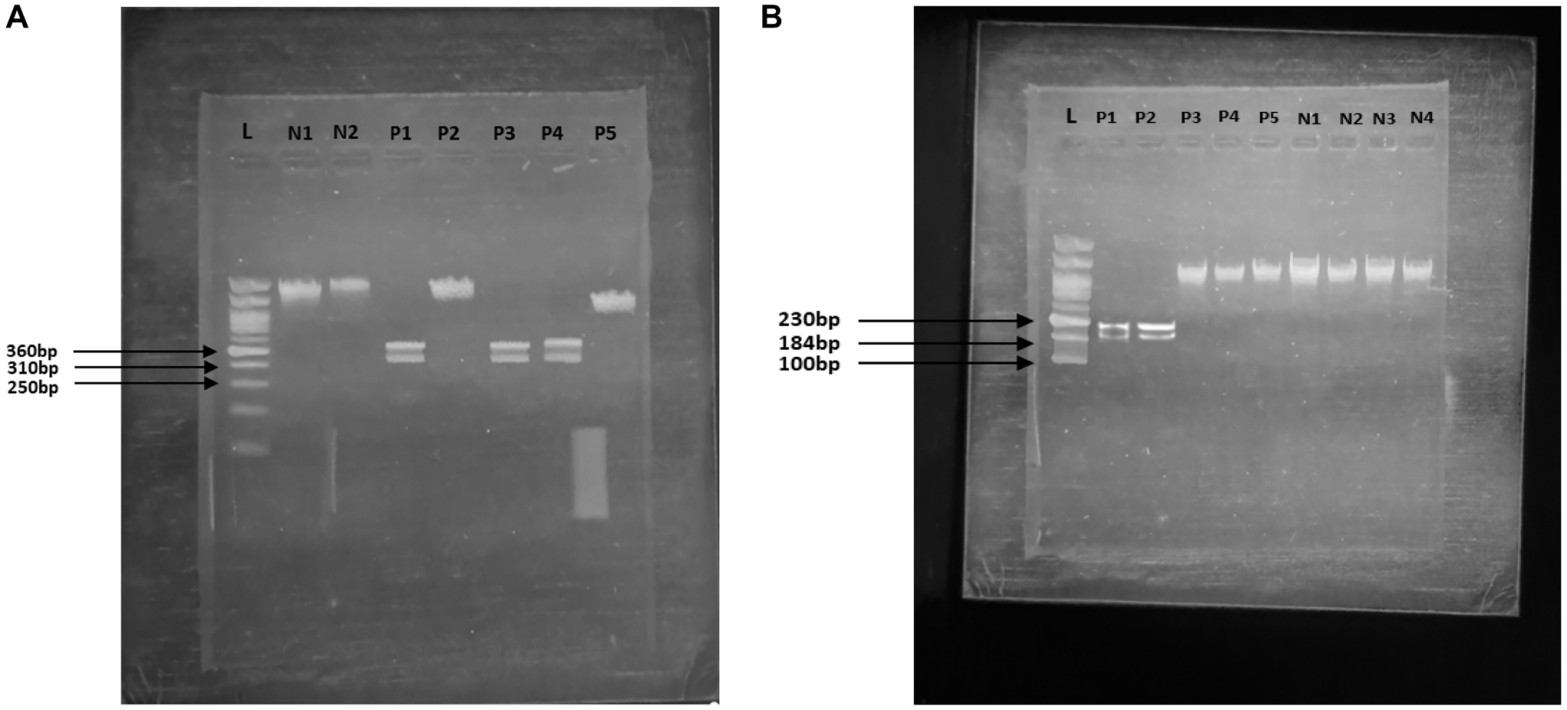
Photograph of agarose gel electrophoresis showing PCR-RFLP of BRCA2 and HOXB13 gene mutation mentioned in Table 1. (**A**) Here P1, P3, and P4 shows the positive results for BRCA2 gene mutation. (**B**) Here P1 and P2 shows the positive results for HOXB13 gene mutation.

Genotyping of the HOXB13 and BRCA2 genes in both prostate cancer cases and controls identified significant associations between specific alleles of these genes and prostate cancer. Also, we did not find any significant results in other genes (BRCA1, RNASEL & ELAC2). The genotyping data showed 39 subjects with mutations of prostate cancer cases from PCR- RFLP analysis (Figure 1), including 26 positives for BRCA2 and 13 positives for HOXB13 mutations. BRCA2 mutations were positive in P1, P3 and P4 (Figure 1A) and HOXB13 mutations were positive in P1, P2 (Figure 1B).

Later allele frequency analysis was performed and shown in the Table 2, revealing that the BRCA2 delT mutation was detected in 26 of cases compared with only 4 of controls, whereas, 66 controls show the T allele compared with only 40 cases, tightening its potential protective role. Thus, BRCA2 gene mutation is strongly associated with an increased risk of developing prostate cancer, as evidenced by the *p*-value (<0.0001) and corresponding confidence interval (3.487 to 32.987).

**Table 2:** Allele frequency analysis of BRCA2 and HOXB13 gene

Gene	Allele	Case	Control	*p*-value	OR 95% CI
BRCA2	T	40	66	<0.0001	10.725 3.487 to 32.987
	delT	26	4		
HOXB13	C	53	70	0.0139	35.5794 2.0684 to 612.0255
	T	13	0		

In the same way, the C allele of HOXB13 was seen in 53 cases and 70 controls, while the T allele was detected only in 13 cases and absent in controls. These values (*P*-value = 0.0139; 95% confidence interval: 2.0684 to 612.0255) indicate significant association with prostate cancer. Collectively, these results provide strong evidence that mutations in BRCA2 and HOXB13 are associated with an increased risk of prostate cancer and highlight their role as potential genetic markers for early detection and risk stratification.

## DISCUSSION

Our research shows that the RFLP-based detection of BRCA2 and HOXB13 mutations can enhance the diagnostic process of prostate cancer, particularly for early detection. These insights are more applicable in settings where complicated genomic testing is not easily available, thereby helping to advance a more widely applicable precision medicine paradigm.

We conducted a cost-effective PCR-restriction fragment length polymorphisms (PCR-RFLP)- based study for genetic predisposition to prostate cancer, with a focus on mutations in important prostate cancer-susceptible genes. Overall, these data suggest a clear relationship between BRCA2 mutation carrier status and prostate cancer risk, as well as genetic variants in HOXB13 and localized prostate carcinoma. Recruitment of further patients also did not reveal significant associations in BRCA1, RNASEL and ELAC2.

Some mutations are also associated with early-onset prostate cancer since prostate cancer cases have a significantly higher average age at diagnosis than controls (57.47 ± 8.94 years vs. 51.27 ± 8.60 years, *p* = 0.0001). The mean PSA level was significantly increased in cases (111.06 ± 214.26) than controls (2.80 ± 1.10) and it is an invaluable diagnostic support to PSA screening. Most of the cases had a Gleason score of ≤ 6 indicating aggressive nature of tumors among mutation carriers. Family history was seen as a major risk factor with 37.88% cases having positive family history versus only 5.71% controls (*p* < 0.0001).

Current genomic technologies like whole-exome and targeted sequencing pave the way for the detection of prognostic and predictive biomarkers such as DNA repair gene mutations (e.g., BRCA1/2, ATM) and changes in the androgen receptor pathway. Although PCR-RFLP does not provide comprehensive genomic information, it is a practical starting point for identifying high-risk mutations in populations where complete genomic profiling is impractical, particularly for BRCA2 and HOXB13. This method allows initial genetic risk stratification and is useful until more comprehensive analysis becomes available through NGS.

Previous studies have already established a link between mutations in BRCA2 and vulnerability to prostate cancer. Kote-Jarai et al. The authors have demonstrated in 2011 that germline mutations of BRCA2 correlate to considerably increased risk of early-onset and aggressive prostate cancer [12]. Similar results were demonstrated in our study where we found the BRCA2 mutation in 26 of 66 prostate cancer patients while only four carry this mutation in control group. The results were consistent among the allele frequencies, confirming BRCA2 had a highly significant *p*-value < 0.0001 (OR of 10.725 with a 95% CI of 3.487 to 32.987). Such a conclusion lends additional support to the assertion that mutations of BRCA2 cause a pre-disposition to prostate cancer, perhaps due to impaired DNA repair function [13].

Likewise, our results suggest HOXB13 mutations are independently associated with prostate cancer risk. The T allele of HOXB13 was observed at 13 prostate cancer cases but was not observed in the controls, leading to a statistically significant association between this polymorphism and prostate cancer (*p* = 0.0139; OR = 35.5794; 95% CI: 2.0684 to 612.0255). This aligns with previous research, including that by Ewing et al. (2012), that found HOXB13 G84E to be a hereditary prostate cancer marker [14]. The role of HOXB13 in both development of the prostate gland and tumor progression indicates that mutations in HOXB13 drive oncogenesis through changes in transcriptional control over key regulatory pathways [15].

Interestingly, our study cohort did not exhibit any major mutations in the BRCA1, RNASEL, and ELAC2 genes. These genes have been implicated in prostate cancer susceptibility in previous studies—as have other MMR pathway genes, although with mixed results [16]. The contribution of BRCA1 mutations to familial prostate cancer is not as strong as that seen for BRCA2, although BRCA1 mutations have been associated with some populations with a family history of prostate cancer [17]. One of these genes is RNASEL, which plays a role in antiviral defense and apoptosis, and has been linked to hereditary prostate cancer in some studies, although this association is controversial, with multiple studies failing to replicate these findings. Likewise, ELAC2 (or HPC2) has been implicated as a candidate gene for prostate cancer risk, but our results are consistent with reports that questioned the clinical relevance of this association in other populations [18, 19].

The use of PCR-RFLP as a cost-effective and targeted genotyping method is one of the major strengths of our study. Next-generation sequencing (NGS) is the standard method for detailed genomic analysis but it is expensive, limiting its adoption, especially in low- resource settings. Our results indicate that PCR-RFLP can be used as an effective method to detect salient prostate cancer-associated mutations, especially in geographical areas with restricted access to relatively advanced genomic technologies. Despite this, the sensitivity of RFLP for detecting rare or novel mutations is inferior to that of NGS, and thus requires further validation in larger and more diverse cohorts.

The use of testing for clinical mutation BRCA1 and BRCA2 is of clinical importance, and it is recommended that mutations be identified in both germline (blood) and somatic (formalin fixed tissue) DNA. Nonetheless, the differences in mutation burden and sensitivity of detection between sample types demand bespoke molecular analytical methods. There is a need to refine testing methods and interpretation policy to adequately evaluate the mutation, as these directly influence clinical action or management. Incorporation of genetic counselling, together with an oncologist, pathologist, and geneticist as a multidisciplinary team, is critical for effective management aimed at patients.

Our study has important clinical implications. BRCA2 and HOXB13 mutations significantly increase the risk of prostate cancer, demonstrating the predictive power of genetic testing for early diagnosis and risk management. Our cohort’s high rate of BRCA2 mutations may make genetic counseling and selective screening for high-risk patients very useful to detect disease at earlier stages and enable personalized treatment approaches. Moreover, combining molecular biomarkers with image modalities (like mpMRI or PET) could enable accurate diagnosis while reducing implementation of invasive biopsy. Notably, the sensitivity to PARP inhibitors and platinum-based therapies is associated with mutations of BRCA2, whereas HOXB13 status may have prognostic value for indolent versus aggressive disease. Incorporation of molecular biomarkers into clinical workflows can enable therapy optimisation based on the patient’s genetic makeup. Geographically distinct clinical settings can enhance patient care through continuously shifting the balance of active and passive pre-intervention risk assessment paired with streamlined treatment pathways. Even periodic genomic assessments, no matter how selective, have the potential to improve the evaluation of risk and optimal treatment course in a constantly evolving clinical setting.

## MATERIALS AND METHODS

The study was conducted in Department of Urology, KLE’s Dr. Prabhakar Kore Hospital and Medical Research Centre Belagavi, between November 2022-January 2025. This prospective study was conducted with the ethical clearance of the Institutional Review Board KAHER/EC/22-23/All male patients both attending Urology department (OPD + admitted patients) presented with clinical features of lower urinary tract symptoms whose serum PSA ≥ 2.5 ng/ml as well as follow up patients who have previous history of treatment of prostate cancer considered in the study as cases. Controls were prospectively recruited from age-matched patients presenting to the urology department with clinical and Laboratory evidence of benign prostate hyperplasia. The study enrolled a total of 136 subjects, including 66 prostate cancer (PCa) cases and 70 patients diagnosed with benign prostatic hyperplasia (BPH) or normal controls. Written consent was taken from all the subjects. Patient demographics and clinical data such as age, PSA levels, and pertinent medical history were collected and analysed for group variability.

Peripheral blood samples were used for extracting genomic DNA using the Invitrogen DNA extraction kit following the manufacturer’s guidelines. Whole blood samples were collected in EDTA tubes and stored at −80°C until processing. The samples were treated with lysis buffer to lyse the membrane and subsequently underwent protein precipitation and removal steps. Ethanol was used to precipitate, wash and rehydrate the DNA in nuclease-free water. DNA was assessed for quality and concentration using a Nanodrop and Spectrophotometer. Agarose gel electrophoresis provided additional assessment of DNA integrity. Targeted gene of interest is amplified using polymerase chain reaction (PCR) using appropriate forward and reverse primers designed using Primer-BLAST tool. Then the amplified product was digested using restriction enzymes which cuts the DNA at recognition sites. Then it is separated on agarose gel electrophoresis to find out the presence of mutation [20].

The Table 3 lists the important prostate cancer related genes with their respective SNPs (rsID), PCR primers for amplification, and restriction enzymes for RFLP analysis. BRCA1, BRCA2, HOXB13, RNASEL, and ELAC2. The five genes known to affect prostate cancer susceptibility are part of the cellular machinery that maintains genomic integrity or contributes to the immune response. Restriction enzymes (BtrI, AccEBI, BfaI, and TaqI) digest specific genes at their designated sites after amplified DNA. If an alteration in the DNA occurs such that the recognition site is changed in a way that the cutting pattern differs (which will typically result in fragments of varying sizes), the results can be visualized by means of gel electrophoresis [20–23]. A cheaper approach was needed to reveal genetic differences in prostate cancer - an alternative to costly sequencing methods.

**Table 3:** List of the genes with respective SNPs, forward primer, reverse primer and restriction enzymes

Gene	Rsid	Forward primer	Reverse primer	Enzy mes
BRCA1	rs869025213	TCAGCTTGACACAGGTTTGG	CTTGATCTCCCACACTGCAA	BtrI
BRCA2	rs80359550	AACGAAAATTATGGCAGGTTGTTAC	CGAAAGGTGAACGACATGATTTAGG	AccEBI
HOXB13	rs9900627	GCTGTCAACTATGCCCCCTT	CTGGTGGGTTCTGTTCTCCC	BfaI
RNASEL	rs486907	GGAAGATGTGGAAAATGAGGAAGA	TGCA GATCCTGGTGGGTGTA	TaqI
ELAC2	rs4792311	GTGAGGGCACATTTGGGCAG	GCACCCTGGCTGCTGTGTTTGT	BfaI

We performed a detailed analysis of clinical data using SPSS software version 25.0. Demographic and clinical characteristics were summarised using descriptive statistics. Group comparisons (cases vs. controls) for age and PSA levels, which are continuous variables, were analysed with an independent samples *t*-test; while categorical variables such as family history and genotype distribution were assessed using Chi-square or Fisher’s exact test. To evaluate the strength of association between gene mutations and prostate cancer risk, odds ratios (ORs) and 95% confidence intervals (CIs) were calculated. A *p*-value of < 0.05 was considered statistically significant.

## CONCLUSION

In summary, our investigation supports a role for BRCA2 and HOXB13 mutations in prostate cancer predisposition and provides reassurance that BRCA1, RNASEL, and ELAC2 are not substantially contributory in this cohort. These results underscore the need for genetic testing for estimated prostate cancer danger and demonstrate the potential applicability of cost- effective, yet high-throughput protocols of polymerase chain reaction restriction fragment length polymorphism (PCR-RFLP) in genetic screening. More studies in larger populations and functional characterization studies will be needed to confirm these results and identify other genetic predictors of susceptibility and progression of prostate cancer.
